# Extrahepatic Drug Transporters in Liver Failure: Focus on Kidney and Gastrointestinal Tract

**DOI:** 10.3390/ijms21165737

**Published:** 2020-08-10

**Authors:** Marek Droździk, Stefan Oswald, Agnieszka Droździk

**Affiliations:** 1 Department of Pharmacology, Pomeranian Medical University, Powstancow Wlkp 72, 70-111 Szczecin, Poland; 2 Institute of Pharmacology and Toxicology, Rostock University Medical Center, 18051 Rostock, Germany; stefan.oswald@med.uni-rostock.de; 3 Department of Integrated Dentistry, Pomeranian Medical University, Powstancow Wlkp 72, 70-111 Szczecin, Poland; agdro@pum.edu.pl

**Keywords:** drug transporters, liver pathology, gastrointestinal tract, kidney

## Abstract

Emerging information suggests that liver pathological states may affect the expression and function of membrane transporters in the gastrointestinal tract and the kidney. Altered status of the transporters could affect drug as well as endogenous compounds handling with subsequent clinical consequences. It seems that changes in intestinal and kidney transporter functions provide the compensatory activity of eliminating endogenous compounds (e.g., bile acids) generated and accumulated due to liver dysfunction. A literature search was conducted on the Ovid and PubMed databases to select relevant in vitro, animal and human studies that have reported expression, protein abundance and function of the gastrointestinal and kidney operating ABC (ATP-binding cassette) transporters and SLC (solute carriers) carriers. The accumulated data suggest that liver failure-associated transporter alterations in the gastrointestinal tract and kidney may affect drug pharmacokinetics. The altered status of drug transporters in those organs in liver dysfunction conditions may provide compensatory activity in handling endogenous compounds, affecting local drug actions as well as drug pharmacokinetics.

## 1. Introduction

Livers, along with kidneys and intestines, are the major organs playing the key role in determination of drug pharmacokinetics, and thus producing an impact on their therapeutic value, namely, clinical efficacy, side effects and drug-drug interactions. Drug transporters located in membranes of hepatocytes, enterocytes and kidney tubule cells belong to main determinants of drug pharmacokinetics. They govern transmembrane movement of drug molecules, and can be subdivided into two major superfamilies, namely, ATP-binding cassette transporters (ABC, consisting of about 50 members, subdivided into 7 families) and solute carriers (SLC, more than 400 membrane proteins grouped into over 60 families). The ABC-transporter superfamily provide efflux functions, which are mediated by the multidrug resistance family (MDR, ABCB), multidrug resistance protein family (MRP, ABCC) and breast cancer resistance protein (BCRP, ABCG2). The SLC carriers are engaged in both cellular influx and cellular efflux of drug molecules, in which the organic anion transporting polypeptide family (OATP, SLC21/SLCO), organic anion transporter family (OAT, SLC22), organic cation transporter family (OCT, SLC22), organic cation/carnitine transporter family (OCTN, SLC22) and multidrug and toxin extrusion protein family (MATE, SLC47) participate.

The ABC transporters and SLC carriers operate in coordinated mode, which allows transmembrane shifting of cation, anion or zwitterion substrates. ABC transporters function primary as active transporters, which transport substrates against their electrochemical gradients, utilizing energy from ATP hydrolysis. The SLC carriers can operate in diverse transport modes. Facilitative SLC transporters shift substrates along electrochemical gradients, and without energy input. The active SLC transporters can mediate transport against a substrate gradient by coupling it to an electrochemical gradient of a co-transported ion (e.g., Na^+^, H^+^) or solute [1].

Cellular uptake of organic cations in the kidney begins with transport on the basolateral surface of the proximal tubular cell, mediated mainly by OCT2, a transporter of organic cations, which takes advantage of the negative potential difference within the cell maintained by the basolateral Na^+^-K^+^-ATPase. On the apical membrane, several carriers (e.g., MATEs) transport organic cations across the apical membrane through electroneutral transport by exchange with proton (H^+^), which capitalizes on the electrochemical gradient that favors movement of H^+^ into the cells. The apical cation transporters list can be complemented by other SLC and ABC transporters (P-gp, BCRP, OCTN1, OCTN2). In the liver, OCT1 is the major organic cation carrier (the function of which is Na^+^-independent, but depends on the electrochemical gradient of the transported organic cation). In hepatocyte cations can also be shifted by OCT3 and NTCP (inward Na^+^ gradient maintained by an Na^+^/K^+^-ATPase is necessary and facilitates the uptake function) [2]. Excretion of cations from hepatocytes can be mediated by ABC transporters, such as P-gp, BCRP or a representative of the SLC family, namely, MATE1 [2]. In the enterocyte, bidirectional cation transport can be facilitated by OCT1, and possibly by OCT3, which acts in concert with export ATP-dependent transporters P-gp and BCRP [2,3,4].

The major transporters and carriers engaged in organic anion transport in humans are MRPs, OATs and OATPs. In the kidney, anions are bidirectionally shuttled by OAT1, OAT2, OAT3 and OAT4 as well as URAT1 (SLC22A12). The principal carriers providing uptake functions, OAT1 and OAT3, are driven by primary active Na^+^ outward transport by the basolateral Na^+^K^+^-ATPase, which causes the gradient facilitating sodium dicarboxylate cotransporters to move Na^+^ and dicarboxylates (such as *α*-ketoglutarate, which also plays a central role in the Krebs cycle (tricarboxylic acid cycle)) inside the cell. The resulting high intracellular concentration of dicarboxylates promotes organic anion uptake across the basolateral membrane in exchange for dicarboxylates. OAT4 and URAT1 facilitate rather apical outward movement of anions. The apical efflux of organic anions in the proximal tubules is provided by the ABC transporters MRP2 and MRP4 [5]. In the liver, anion transport is facilitated mainly by Na^+^-independent transporters OATP1B1, OATP1B3, OATP2B1 as well as OAT2. The transport mechanism of OATPs remains unclear, and among the postulated mechanisms are enumerated bicarbonate as counter-ion, bicarbonate efflux coupled transport or glutathione gradient [6]. The driving force for OAT2 is not well established [7]. Biliary and basolateral efflux of organic anions in hepatocytes is mediated by energy-dependent ABC transporters MRP2, MRP3, and MRP4 as well as by the SLC family representative MATE1 [2,4]. Enterocyte possesses a battery of transporters/carriers, which enable cation handling. Uptake functions can be provided by OATP2B1 function, possibly driven by proton gradient (H^+^) [8]. Some controversies remain as to the intestinal expression of OATP1A2 [9]. The function of ABC transporters enables organic anion efflux from enterocytes to the gut lumen (MRP2) or across the basolateral membrane (MRP1, MRP3, MRP4) [2].

Few of the drug transporters/carriers are considered as important in clinical practice. The list of transporters of clinical relevance include: P-glycoprotein (P-pg, *ABCB1*), breast cancer resistance protein (BCRP, ABCG2), organic anion transporter (OAT) OAT1 (*SLC22A6*) and OAT3 (*SLC22A8*); organic cation transporter (OCT) OCT2 (*SLC22A2*), and multi-drug and toxin extrusion protein (MATE) MATE1 (*SLC47A1*) and MATE2-K (*SLC47A2*), as well as organic anion transporting polypeptide (OATP) OATP1B1 (*SLCO1B1*) [10]. The same drug transporters are listed by the FDA for evaluation in drug-drug interaction studies [11]. The present review mainly focuses on the abovementioned transporters.

**Figure 1:**
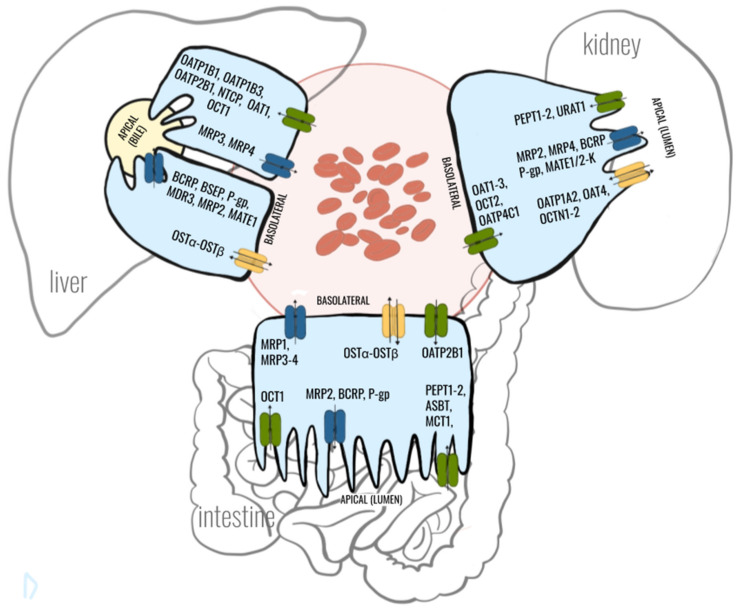
Major membrane drug transporters expressed in hepatocytes, enterocytes and proximal tubule kidney cells.

Principal cells of the major organs engaged in drug pharmacokinetics possess a specific battery of drug transporters and carriers engaged in drug transport (Figure 1). Human hepatocyte uptake transporters in the basolateral (sinusoidal) membrane comprise three members of the OATP family: OATP1B1, OATP1B3 and OATP2B1 (*SLCO2B1*) as well as two members of the SLC22A family: OCT1 (*SLC22A1*) and OAT2. Efflux pumps in the hepatocyte located in the basolateral membrane include multidrug resistance-associated proteins: MRP3 (*ABCC3*), MRP4 (*ABCC4*) and MRP6 (*ABCC6*). The apical (canalicular) efflux pumps of the hepatocyte include P-gp, bile-salt export pump (BSEP, ABCB11), BCRP and MRP2. In addition, multidrug and toxin extrusion protein 1 (MATE1; *SLC47A1*) is located in the apical hepatocyte membrane [3]. The most abundant transporters expressed in the liver are uptake carriers, namely, OATP1B3, OATP1B1, OCT1 and OATP2B1 (mediating the uptake of endogenous compounds like bilirubin and bile salts), whereas MATE1, P-gp, BCRP and MRP2 are the least present [12,13].

The enterocyte apical (luminal) membrane hosts uptake carriers, namely, OCT3, peptide transporter 1 (PEPT1, *SLC15A1*), apical sodium-dependent bile acid transporter (ASBT, *SLC10A2*) and monocarboxylic acid transporter 1 (MCT1, *SLC16A1*) as well as efflux function transporters, namely, P-gp, BCRP and MRP2. The basolateral membrane of intestinal epithelia harbors heteromeric organic solute transporter functioning as bidirectional carrier (OSTα-OSTβ, SLC51), as well as MRP1 and MRP3 as uptake system or OATP2B1 providing uptake activity. Some controversies exist as to membrane localization of OCT1 and OATP2B1, but the most recent findings provide evidence for the apical (OCT1) and basolateral (OATP2B1) expression [14,15,16]. It should also be stated that drug transporter distribution in specific functional segments of the gastrointestinal tract is not homogenous. Our human data suggest that the most abundant transporters in the duodenum are MCT1, PEPT1 and MRP3; in the jejunum they are PEPT1, MCT1, P-gp, and MRP3; in the ileum they are PEPT1, P-gp, ASBT, MCT1, and BCRP; and in the colon they are MCT1, MRP3, and MRP4 [4,17].

The kidney tubule (proximal is the best defined) cells are endowed in the apical (luminal) membrane with OAT4 (SLC22A11), urate transporter 1 (URAT1, *SLC22A12*), PEPT1, PEPT2 (*SLC15A2*), MRP2 and MRP4, MATE1 and MATE2-K (*SLC47A2*), P-gp, organic cation/ergothioneine transporter (OCTN1; *SLC22A4*) and organic cation/carnitine transporter (OCTN2; *SLC22A5*). Basolateral uptake transporters in proximal tubule epithelia include OATP4C1 (*SLCO4C1*); OCT2; and OAT1, OAT2 and OAT3 (*SLC22A8*) [14]. In the human kidneys drug transporters belonging to SLC families (OCTs, OATs, MATEs) are more abundantly expressed than transporters belonging to the ABC-family. OAT1, OAT3, OCT2 and MATE1 are the most abundant renal SLC transporters while MDR1, MRP1 and MRP4 are the dominating ABC transporters [18,19]. A functional segment specific transporter panel expression, as in the gastrointestinal tract, in proximal, distal and collecting duct cells is suggested. However, most of the available information about drug transporters is confined to proximal tubule cells. OCT2 is an example of the transporter exclusively expressed in proximal tubule, and MRP3 in distal tubule cells [19,20].

## 2. Crosstalk between Gut-Liver-Kidney-Axis in Context with Drug Transporters

The gut-liver-kidney forms a group of functionally coordinated organs, referring especially to drug transporters and drug metabolizing enzymes. Rosenthal et al. [21] analyzed a network from a randomly selected set of 690 genes in the liver, kidney and gastrointestinal tract, and evidenced that ABC transporters, SLC carriers and drug metabolizing enzymes form the strongest cluster of inter-connected genes. When only single sets were considered (i.e., just SLC genes or just ABC genes) or a set of ABC and SLC genes a smaller fraction of the highest ranking edges still mapped to the gut, liver and kidney. It suggests that the membrane transporters and carriers in these organs can function in coordinated manners. In this regard, liver dysfunction, especially in advanced stage, results in liver products (bilirubin, bile acids/salts) and microbiota toxin accumulation in plasma, which in turn may affect the function of other organs, including the kidney and gastrointestinal tract. It can be assumed, that kidney and gastrointestinal tract ABC transporters and SLC carriers are exposed to the action of the toxins, and according to the remote sensing and signaling hypothesis, mediate communication between organs and the host organism and gut microbiota. Among the proposed common regulators of the gene expression regulation, transcription factors/nuclear receptors, hepatocyte nuclear factor (HNF)-1α, HNF-4α and pregnane X receptor (PXR) were proposed, and thus appear to be central nodes in the gut-liver-kidney network [21]. The strongest mechanistic link was established between the transcription factor HNF4α and MRP2, an important transporter of endogenous organic anions in the liver, kidney and intestine [22]. It accepts bilirubin mono- and bisglucuronosyl conjugates as well as salts (taurocholate, taurochenodeoxycholate sulfate and taurolithocholate sulfate). Similarly, the remote sensing and signaling network captures most transporters involved in various aspects of bile acid handling in different organs, including MRP2, BSEP, OSTα, OSTβ, OATP1B1, NTCP, ASBT and OAT3 [23,24]. Bile salts induce the activity of transcriptional repressor—small heterodimer partner (SHP)—and thus interfere with transactivation mediated by nuclear receptors, for example, HNF-1α, HNF-4α, FXR and PXR [25,26]. This mechanism allows coordinated adaptation of the organs to bile salts handling. The list of other transporters regulated by HNF-1α and HNF-4α include OCT1, OCT2, OAT1 and OAT2, which recognize endogenous small molecules, including bile acids (OCT2), creatinine (OAT2, OCT3) or uric acid (OAT1) [2]. Thus, liver failure, associated with accumulation of bile acids, can lead to modulation of the SLC22A transporter functions, providing functional adaptation to liver dysfunction.

To other potential regulators of drug transporter expression in liver failure, especially in more advanced stages, inflammatory mediators, for example, tumor necrosis factor α (TNF-α), interleukin-1β or IL-6 belong, in which increased systemic levels are observed in cirrhotic patients [27,28]. Regulatory effects of those cytokines has been documented in several studies. It was demonstrated that TNF-α, by reducing the activity of CAR and PXR, can trigger downregulation of *Abcc2*/Mrp2. Reduced activity of PXR was also implicated in the downregulation of *Abcc2* and *Bsep* mediated by IL-6 in the liver [29]. Interleukin-1β was shown to reduce expression of slc*10a1* via a decrease of HNF-1α binding to its promoter as well as *Abcc2* via reduction of the activity of FXR, CAR, and PXR [29].

Not only bile acids/salts and general inflammation, which altered systemic status, is observed in liver pathologies, but also products of disturbed gut microbiota or altered handling of bile acids by intestinal bacteria, may affect expression and function of the transporters in the liver, kidney and intestine [30]. However, mostly experimental data are available. A model of dysbiotic mice (germ-free mice) demonstrated that the dysbiotic state entailed significant decrease in liver and kidney Oatp1a1 levels as well as Bcrp1 and Oct1 in the liver. This study provides evidence that alterations of intestinal flora might markedly influence the protein expression of transporter molecules in the liver and kidney [31]. The proposed mechanism underlying the observed changes in the transporter expression involves reduction of lithocholic acid levels (a secondary bile acid produced by intestinal bacteria) due to dysbiosis, with consequent reduced activation of PXR and farnesoid X receptor (FXR), which are involved in positive regulation of the transporters [32]. Likewise, products of intestinal bacteria metabolism can influence expression of transporters in remote organs, as it was evidenced in the kidney. The kidney proximal tubules, by sensing elevated endogenous gut microbiome, derived metabolite indoxyl sulfate, and can regulate body homeostasis by stimulating secretory mechanisms through OAT1 [33]. The metabolite sensing and signaling mechanism in the kidney resulting in OAT1 upregulation was documented to be regulated by the interplay of the aryl-hydrocarbon receptor (AhR)-ARNT complex and downstream EGFR MAPK-ERK pathways. The reported studies suggest the existence of the network of drug transports in remote organs (e.g., gut−liver−kidney axis) regulated in coordinated fashion by metabolites and signaling molecules, which tune the expression and function of those drug transporters accordingly.

## 3. Effects of Liver Failure on Hepatic Drug Transporters

Liver pathology produced by different underlying processes (e.g., genetic, cholestatic, toxic, viral, metabolic, autoimmune) results not only in hepatic dysfunction reducing, with the disease progression and developing fibrosis, the functional mass of hepatocytes, but also in disturbed organ architecture, also affects the functions of many other organs to which many mechanisms may contribute, namely, reduction in portal (affecting gastrointestinal tract) and renal blood flow, glomerular filtration rate, serum albumin concentration, circulating bile acids/salts, reduced hematocrit value or hypoxemia [34,35,36,37].

It is obvious that liver pathologies themselves also affect expression and function of drug transporters in the liver. A better understanding of the impact of liver diseases on expression of drug transporters is not only important to better characterize drug pharmacokinetics, intrahepatic drug actions or drug-drug interactions, but also to understand the organ pathophysiology (membrane transporters are also involved in the transport of endogenous substrates).

The very limited available information suggests that the expression of drug transporters and carriers in the liver depends on the stage of the organ dysfunction. The results of our proteomic study suggests that in the course of gradual deterioration of liver function, up-regulation of efflux transporter P-gp as well as induction of MRP4 expression are produced. MRP2 is the downregulated efflux transporter. On the contrary, the function of uptake carriers, namely, NTCP, OCT1, OATP1B1 and OATP2B1 decrease [13]. The decline in levels of the transporters implicated in bilirubin (the uptake transporters OATP1B1 and OATP1B3 and the efflux transporter MRP2) and bile salts handling correspond with the observed concentration changes of these endogenous transporter levels in liver failure [38]. Deterioration of liver function entails an increase in bilirubin blood concentrations, due to reduced hepatic uptake (OATP1B1 and OATP1B3) and sinusoidal elimination (MRP2) [39]. On the other hand, OATP1B1, OATP1B3, NTCP, P-gp, MRP2 and MRP4 are involved in the handling of bile salts [40,41]. Reduced levels of the canalicular transporter MRP2, accompanied by decreased abundance of basolateral (sinusoidal) uptake transporters (OATP1B1, OATP1B3, and NTCP) and increased values of efflux transporters (P-gp, MRP4) most likely represent adaptive changes of hepatocytes providing a defense mechanism against bile salts in hepatic insufficiency.

The drug transporter levels may also be affected by a specific liver pathology. Most of the available information is derived from studies presenting expression (mRNA levels) and semiquantitative protein (Western blot, immunocytochemistry) data. However, more precise proteomic quantitative information (mass spectrometry based methods) is also available. Those reports indicate no correlation between expression and protein abundance in the case of many transporters [13,42,43,44,45,46,47]. Therefore, findings based only on transcriptome analysis may not reflect the real status of transporter abundance. However, proteomic studies suggest that drug transporter changes are rather associated with a specific underlying pathology, not with just deterioration of liver function. From the available information, although still scarce, some general conclusions can be established. It seems that parenchymal liver damage (hepatitis C, alcoholic liver disease, nonalcoholic fatty liver disease) are rather associated with dysfunction of the uptake systems. In the studies, downregulation of basolateral uptake carriers, namely, NTCP, OATP1B1, OATP1B3 and OCT1 is seen. From the efflux transporter changes, the studies rather point to downregulation apical of MRP2 and BSEP [13,42,44,47]. The observations from primary biliary cholangitis and primary sclerosing cholangitis, demonstrate that in cholestatic liver diseases, efflux changes of both apical and basolateral transporters are rather seen. The abundance of the canalicular P-gp transporter as well as the basolateral MRP4 efflux pump are increased, whereas levels of basolateral uptake system carriers (NTCP, OATP1B1, OATP1B3) are maintained [13]. Autoimmune hepatitis is characterized by drug transporter changes resembling the panel of alterations observed in cholestatic diseases, mainly as changes in efflux transporters are present, in other words, upregulation of P-gp and MRP2 and downregulation of MRP2 [13]. As stated above, the changes in the abundance of the uptake and efflux transporters may represent a protective adaptation of hepatocytes against damage produced by bile salts and bilirubin.

The pathophysiology of liver failure depends on the primary mechanisms engaged in the destruction of hepatocyte function and the organ structure. It also seems to be mirrored by different statuses of drug transporters in the organ, as stated above, characteristic for parenchymal and cholestatic liver diseases. The existing evidence suggests that the liver pathology affects not only levels of the transporters in the diseased organ, but also in the gastrointestinal tract and kidneys, and thus creates a complex network which may affect drug pharmacokinetics and drug-drug interactions.

The available experimental and clinical information provides evidence that not only liver failure (being presented in the current review), but also other pathological states may affect levels and function of drug transporters, namely, kidney failure, hyperthyroidism, hyperparathyroidism, obesity, diabetes mellitus, systemic inflammation and Alzheimer’s disease as well as gastrointestinal pathology (inflammatory bowel disease, celiac disease, cholestasis) and kidney pathology (drug induced acute kidney injury, chronic kidney disease, glomerulonephritis) themselves [34,48,49].

## 4. Effects of Liver Failure on Drug Transporters in the Gastrointestinal Tract

Disturbed liver architecture during the development of cirrhosis leads to reduced blood flow via the portal vein, and thus in intestinal congestion and portal hypertensive gastropathy. Consequently, various complications of the gastrointestinal tract are seen in advanced liver diseases to which low serum albumin, high bilirubin and bile salts levels, low platelet or erythrocyte counts and hypoxemia may contribute [50].

Changes in drug transporters’ expression in the gut observed in liver dysfunction states may represent adaptation mechanisms involved in bile acid, bile salts or other molecules handling, and support the remote sensing and signaling hypothesis. The available information from experimental studies, and to a lesser extent from clinical observations, document the impact of liver dysfunction on the transporters’ levels in the gastrointestinal tract (Table 1).

### 4.1. In vitro Studies

In vitro experiments carried out mostly in Caco-2 cells (human colon carcinoma cell line) suggest downregulation of the expression and function of *ABCB1*/P-gp. Exposure of the cells to plasma from animals with acute liver failure (ALF) induced by administration of carbon tetrachloride (CCl4) [51] and thioacetamide [52] proved its significantly greater inhibitory potency on P-gp-mediated rhodamine-123 transport across Caco-2 cell monolayers than plasma from control rats. Contrary to significant reduction in P-gp levels, the plasma from thioacetamide-induced ALF rats did not produce a significant impact on Caco-2 expression of BCRP [52].

### 4.2. Animal Models

Animal studies in rats with CCl4-induced ALF confirmed the abovementioned findings in Caco-2 cell cultures since significantly reduced intestinal P-gp transporter function of digoxin and rhodamine-123 (absorption and exsorption substrates) was found. However, Western blot analysis demonstrated an unchanged abundance of intestinal P-gp in ALF. In contrast, the results of ex vivo everted intestine study in ALF rats suggested a significantly higher efflux transport rate of the P-gp substrates [53]. So, this set of experiments demonstrated differential effects of ALF on P-gp function in in vivo and ex vivo conditions. Contrary to parenchymal liver injury, the cholestatic model did not evidence its impact on P-gp levels. It was found that P-gp protein abundance in the rat ileum was not affected by mechanical cholestasis induced by bile duct ligation (BDL) during the seven days post-surgery [54].

Intestinal changes in Bcrp levels may provide the adaptive response to cholestasis, and increased exposure to bile salts of hepatocytes and other cell types. The study of Mennone et al. [54] found that bile duct ligation (BDL; experimental model of obstructive cholestasis) resulted in a significant increase in Bcrp protein expression in the ileum by seven days. Bcrp transporter exports bile acids into the gut, and thus in cholestatic state its increased intestinal levels may serve as a protective mechanism against excessive exposure to systemic bile acids. This study also documented that Bcrp levels in the liver and kidneys were downregulated by BDL in mice.

The CCl4-induced ALF with hyperbilirubinemia rat model study demonstrated a decrease of Mrp2 protein levels in the jejunum to 41% of the controls’ abundance. However, it should be stated that the impact of the liver failure on Mrp2 abundance was not observed in the ileum [57]. The changes of the transporter protein abundance resulted in reduced (up to 31% of controls) Mrp2-mediated transport of 2,4-dinitrophenyl-S-glutathione (DNP-GSH; Mrp2 substrate) in the jejunum in vitro. However, ALF did not affect DNP-GSH efflux rate in the ileum. These findings suggest that Mrp2 function may be differentially regulated in specific functional sections of the gastrointestinal tract. Similar results, regarding intestinal levels of Mrp2, to CCl4-induced ALF were reported in BDL rats. Dietrich et al. [55] found significant downregulation of Mrp2 mRNA and protein levels in rats with biliary obstruction (but not biliary depletion). Similar findings on Mrp2 transporter, but only at mRNA level, in the small intestine of BDL rats were reported by Kamisako et al. [58]. However, this study also revealed time-dependent changes in mRNA levels, as a remarkable decrease was observed 24h after BDL, with subsequent recovery 72h after BDL.

The BDL rats also demonstrated no effects of cholestasis on small intestinal Mrp3 mRNA expression up to 72h after the surgery [58].

The same model of mechanical cholestasis, namely, BDL rats, revealed marked downregulation of both Osta and Ostb protein levels as well as insignificant elevation of Asbt in the gastrointestinal tract [57]. These observations are in keeping with FXR-mediated feedback expression regulation of these transporters by bile acids. It was demonstrated that FXR activation by bile acids reduced the expression of ASBT, which mediates bile acid transport from the intestinal lumen into the enterocytes, whereas it increased the expression of OSTs, thus eliciting intracellular trafficking of bile salts from the apical to the basolateral membrane. The observed changes in Asbt, Osta and Ostb document adaptive mechanisms in enterocytes to the absence of bile acids produced by BDL.

### 4.3. Clinical Studies

From the results of clinical observations regarding ABCG2/BCRP levels in the duodenum in patients with obstructive cholestasis, Zimmermann et al. [56] reported downregulation to 53% of the mRNA expression and protein staining reduced to 57%. However, unchanged duodenum BCRP protein levels were found by Dietrich et al. [55].

Information about MRP2 expression and protein abundance in humans with obstructive cholestasis indicate reduction (27.3%–20.3% of control patients) in duodenum MRP2 protein expression. The authors also reported that this reduction correlated with the duration of cholestasis and was reversible after reconstitution of bile flow by stenting of the common bile duct. However, no significant differences in MRP2 mRNA levels were detected [55].

Clinical observations in patients with obstructive cholestasis also revealed that intestinal protein expression of P-gp and MRP3 was unchanged [55].

From the available in vitro, animal and scarce clinical data (Table 1) depicting the changes in ABC transporters and SLC carriers in the gastrointestinal tract under liver failure conditions, it is seen that most of the transporters behave in a similar way, and experimental models can be useful in explanation of drug pharmacokinetics in patients with liver dysfunction.

## 5. Effects of Liver Failure on Drug Transporters in the Kidney

Among numerous systemic effects associated with deteriorating liver failure and cirrhosis, disturbed kidney function is observed. Renal diseases accompanying liver failure comprise mainly two major categories as for etiology, namely, hepatitis virus-associated nephropathy including membranous nephropathy, membranoproliferative glomerulonephritis and mesangioproliferative glomerulonephritis as well as hepato-renal syndrome (HRS) [50]. HRS may be classified into two types: type-1, characterized by a rapid progression of renal failure (within two weeks) usually precipitated by a spontaneous bacterial peritonitis; and type-2, of less severe course. HRS is mainly related to renal vasoconstriction following a reduction of effective circulating volume due to peripheral vasodilation [59]. However, up to now there is a lack of published information about renal drug transporter changes in patients with kidney failure precipitated by liver dysfunction/cirrhosis.

Likewise to the gastrointestinal tract, experimental findings suggest the existence of adaptive changes in the kidney to molecules accumulated in the body during liver dysfunction. In this regard, ABC transporters and SLC carriers in the kidney are exposed to the action of the toxins, which according to the remote sensing and signaling hypothesis, mediate communication between organs [60] (Table 2).

### 5.1. In vitro Studies

The effects of synthetic conjugated bilirubin, sulfate-conjugated bile acids, human bile and unconjugated bilirubin on human MRP2 expression in cell lines were reported by Tanaka et al. [62]. An increase in mRNA expression of *ABCC2* in human renal proximal tubular epithelial cells (RPTEC) after treatment with conjugated bilirubin, sulfate-conjugated bile acids or human bile was observed.

Proximal tubular cells freshly isolated from rat kidneys harvested from rats with obstructive cholestasis induced by a 24-h bile duct ligation demonstrated a significant decrease of maximum transporter velocity (V_max_) of Asbt, indicating a downregulation of the transporter function in cholestasis. The functional downregulation occurred without a change of the Asbt protein content of the proximal tubular cells [67]. However, it should be stated that due to functional differences between human (RPTEC) and rat proximal tubular cells (FPTC) direct comparison/data extrapolation might not be possible.

### 5.2. Animal Models

An upregulation in *Abcc2*/Mrp2 (mRNA, protein) in kidneys starting from one day after BDL in rats was reported by Lee et al. [64]. Tanaka et al. [62] not only confirmed increased Mrp2 protein levels and mRNA expression in the kidneys 24 h after BDL (in the liver *Abcc2*/Mrp2 protein and mRNA expression were downregulated), but also reported an increased renal Mrp2 function assessed via PAH clearance in a rat model of obstructive jaundice. Those results suggest that the increased renal Mrp2 levels might constitute an alternative pathway for bilirubin conjugate excretion during obstructive cholestasis. Similar observations also come from rats with parenchymal liver damage. Significantly higher mRNA expression of Mrp2 in the kidneys of rats with liver failure exposed to CCl4 was reported by Khemawoot et al. [63]. The transporter increased expression was associated with increased kidney elimination of valproic acid (a substrate for Mrp2). Another BDL rat study complemented data on OATs in the kidney. Immunoblotting showed a significant increase in the abundance of both OAT1 and OAT3 in homogenates from the renal cortex. In basolateral membranes from the kidney cortex of BDL rats, OAT1 abundance was also increased, whereas OAT3 levels were not modified [69]. The study of Denk et al. [66] in BDL rats provided information about significant decrease in Mrp4 in kidney 14 day after the ligation. However, real-time-PCR demonstrated no major changes of Mrp4 mRNA levels in kidney after BDL.

Another cholestatic model, in other words, Eisai hyperbilirubinemic rats (EHBR), which lack functional Mrp2, revealed induction of Oat3 protein in the kidney, whereas Oat1 levels did not change [71]. The same experimental model (EHBR) also evidenced significant upregulation of Mrp3 and Mrp4 expression and protein abundance in the kidneys, where Mrp3 protein was localized at the basolateral membrane and Mrp4 demonstrated apical localization [65,68].

Likewise to liver cholestasis, dysfunction of hepatocytes related to nonalcoholic steatohepatitis (NASH) affects renal expression of drug transporters. Animal models of NASH, namely, the methionine and choline-deficient (MCD) diet, atherogenic diet, fa/fa rat, ob/ob and db/db mice demonstrate altered statuses of both ABC transporters and SLC carriers [61]. From the models studied, an upregulation (mRNA and protein levels) of efflux transporters is observed. Mrp2, Mrp4 and P-gp in the rat MCD model as well as Mrp2 in the ob/ob mice were elevated in comparison to control animals. Bcrp expression and protein abundance were not affected by NASH. In contrast to efflux transporters, uptake transporter mRNA expression and protein abundance were generally rather stable in NASH models. Oat1 and Oct1 renal uptake transporters expression was not significantly changed at the protein level in the rat models and Oat1 also in the db/db model. Oatp1 protein level was significantly reduced in the mouse ob/ob and db/db models. Oct2 was studied only at mRNA level, and was shown to be downregulated in the MCD, ob/ob, and db/db mice along with an induction seen in the atherogenic model. Induction of renal mRNA expression of Oat3 in the rat MCD as well as the mouse ob/ob and db/db models was also noted. 

### 5.3. Clinical Studies

Currently no direct clinical data on drug transporter expression and function in the kidney from humans with liver dysfunction are available.

The current, however only experimental, data demonstrate the relevance of renal elimination of drug transporters substrates as an alternative pathway, under conditions of impaired hepatobiliary function, as occurs in parenchymal liver damage or obstructive cholestasis, for excreting those toxic compounds that the liver could not eliminate from the body by itself [62,63]. 

## 6. Pharmacokinetics and Drug Actions

Adaptive changes in transporter expression and function during liver dysfunction observed in the gastrointestinal tract and the kidney originate from interorgan communication mediated by endogenous compounds or products of intestinal bacteria metabolism. Both animal and clinical studies provide evidence that changes in transporters’ statuses affect the kinetics of drug molecules recognized by ABC transporters and SLC carriers. However, there is relatively limited number of model drugs, which are rather unique transporter substrates, and are not subjected to metabolism (which if fact is the next very important player in drug pharmacokinetics). However, animal model studies allowed evaluation of drug transporters’ impacts on model drug/compound handling, and thus provide direct evidence that altered transporter function during liver failure influence drug handling in other organs.

Cholestatic models, in other words, BDL animals, revealed the impact of Mrp2, Oat1-3 and Oct2 on their substrate transport. It was revealed that oral bioavailability of Mrp-2 substrate (and food-derived carcinogen 2-amino-1-methyl-6-phenylimidazo(4,5-b)pyridine (PhIP)) was elevated 2.5 times in comparison with control, sham-operated rats. These changes in PhIP handling can be ascribed to downregulated Mrp2 levels, and thus efflux function, in rat gastrointestinal tracts [55]. As it was shown in other BDL studies, liver Mrp2 levels are downregulated, which may lead to less efficient PhIP biliary excretion, and thus increased bioavailability. However, other studies point out increased Mrp2 levels in the kidney (as compensation of deficient Mrp2 function in the liver), which may contribute to more efficient PhIP urine excretion [62,64]. It may be speculated that the kidney compensation of deficient liver Mrp2 levels was not sufficient to balance disturbed excretory functions of the liver. Clinical observations on MRP2 substrates may reflect results of the experimental studies. The pharmacokinetics of small molecule chemical drugs such as valsartan and telmisartan may be altered. A significant increase in area under the curve (AUC) and maximum plasma drug concentration (C_max_) of valsartan and telmisartan (MRP2 substrates) were increased by 2.2-fold/1.8-fold and 2.4-fold/6.5-fold in patients with liver dysfunction, respectively [72,73]. To date, sartans are also substrates of OATPs and the carrier’s downregulation observed in liver disease (as discussed above) can contribute to decreased hepatic uptake and biliary elimination (and thus increased bioavailability).

Another example of studies which may corroborate with human pharmacokinetics studies refer to OATPs. Downregulation of liver OATPs was demonstrated in many clinical studies, and was used to explain pharmacokinetic data, which revealed increased concentrations of OATPs’ substrate rosuvastatin (also BCRP substrate) in patients with mild hepatic impairment compared with healthy controls along with changes in relevant pharmacokinetic parameters: AUC 1.2-fold; C_max_ 2.1-fold [74]. Animal observations can add another possible contributing factor, which is a decreased Oatp1 levels in the kidney shown in the animal NASH model of liver dysfunction, and thus reduced capacity of Oatp1 substrate renal excretion [61]. These observations are also in line with information about bromosulfophthalein (BSP, a prototypical organic anion) kidney elimination in BDL rats [75]. The increase in the renally secreted load of BSP might be explained by the higher measured expression of Oatp1 observed in apical membranes from kidneys of BDL animals.

However, animal data are not always in line with clinical observations, highlighting interspecies differences. Rat studies revealed induction of Oct2 in the kidney after bile duct ligation, which was associated with increased clearance of its substrate, namely, cimetidine [76]; whereas human pharmacokinetic studies revealed significantly decreased renal clearance (and significantly reduced total body clearance) of the drug in cirrhotic patients [77]. 

The model substrates of OATs were not evaluated in patients with liver dysfunction, but there is information from animal studies suggesting that their dysfunction in subjects with liver failure might impact handling of molecules recognized by the transporters. The studies on p-aminohippurate (PAH, a substrate for OATs) in rodent cholestatic models revealed that downregulation of basolateral levels of Oat1 was associated with a reduction in the renal clearance and in the excreted and secreted load of PAH [70] (in fact this study demonstrated stable levels of Oat1 protein in kidney homogenates, and decreased in the basolateral membranes, as well as increased abundance of Oat3 protein in both kidney cortex homogenates and in basolateral membranes; and so the results suggest the dominant role of Oat1).

Animal study in BDL rats also demonstrated upregulation of Oat1 and Oat3, which might explain, at least in part, the increased systemic and renal elimination of furosemide (OATs substrate) [69]. Clinical observations may suggest increased function of OATs in patients with alcoholic liver disease. It was documented that furosemide induced natriuresis strongly correlated with urinary furosemide concentrations, which was decreased in the patients. Combining experimental data, it may be stated that decreased OAT1 and OAT3 expression in the patients with liver dysfunction can lead to reduced furosemide transport via OAT1 and OAT3 to its site of action, and thus decreased natriuteric effects. The reported plasma furosemide concentrations were higher in patients with reduced furosemide excretion and impaired natriuresis, thus supporting the defective furosemide transport into the tubule [78].

The knowledge about drug transporter functions can be translated into practical clinical applications. In clinical practice antagonists of some membrane transporters are applied, in other words, in the treatment of diabetes mellitus the drugs (gliflozins) target the sodium-glucose cotransporter 2 (SGLT2, SLC5A2) in the kidney and prevent the reabsorption of glucose, or in patients with gout lesinurad, an inhibitor of URAT1 (SLC22A12) carrier is used to selectively inhibit uric acid reabsorption in the kidney [79,80]. The gastrointestinal transporter function is also used to increase absorption of drugs. The PEPT1 transporter, which mediate transport of di/tripeptides and peptide-like drugs (ACE inhibitors or β-lactam antibiotics) is used to increase oral bioavailability of poorly absorbable drugs, which are linked to structural motifs recognized by PEPT1 (Trojan horse strategy). This approach was applied to increase intestinal absorption of acyclovir (by binding it to L-valine—valacyclovir), oseltamivir or cefuroxim-axetil [81].

## 7. Conclusions

In conclusion it can be stated that liver dysfunction affects ABC transporters and SLC carriers’ levels and functions not only in the diseased organ, but also in remote tissues, as in this review in the gastrointestinal tract and the kidneys. The changes of the transporter and carrier functions are operating in concert in different organs adapting to alter the functional state of the organism. Understanding transporter functions can not only allow better prediction of drugs’ pharmacokinetics, drug-drug interactions, drug-endogenous substrates interactions and biological effects (other dysregulated mechanisms in liver pathologies can also affect drug pharmacokinetics, to date altered function of drug-metabolizing enzymes, reduction in portal and renal blood flow, glomerular filtration rate, serum albumin concentration, circulating bile acids/salts, hematocrit value and functional liver mass can be found among potential modifiers [56,59]), but also point out potential therapeutic targets to control various underlying pathologies, as many drug transporter endogenous substrates were defined. It can be added that a lot of orphan transporters with unknown substrates or functions are annotated in the human genome. The characteristics of these transporters, namely, tissue/organ distribution and expression, subcellular localization and endo- and exogenous substrates, still remain to be defined. As discussed in this review, information about transporters can be of significant pathophysiological and therapeutic value, and constitute a background for the treatment of various diseases.

## Figures and Tables

**Table 1:** Effects of Liver Failure on Expression and Function of Drug Transporters in the Gastrointestinal Tract (Details in the Text).

Transporter	Cell Models (ref)	Animal Studies (ref)	Clinical Studies (ref)
P-gp	↓ Caco-2 [51] ↓ Caco-2 [52]	↓ CCl4-ALF rats [53] ↑ CCl4-ALF rats [53] ↔ BDL rats [54]	↔ cholestasis [55]
BCRP	↔ Caco-2 [52]	↑ BDL rats [54]	↓ cholestasis [56] ↔ cholestasis [55]
MRP2		↓ ↔ CCl4-ALF rats [57] ↓ BDL rats [58] ↓ BDL rats [55]	↓ cholestasis [55]
MRP3		↔ BDL rats [58]	↔ cholestasis [55]
ASBT		↔ BDL rats [57]	
OSTα		↓ BDL rats [57]	
OSTβ		↓ BDL rats [57]	

ALF: acute liver failure; BDL: bile duct ligation; Caco-2: human colon carcinoma cell line; CCl4: carbon tetrachloride; ↓: decreased levels; ↑: increased levels; ↔: levels not changed.

**Table 2:** Effects of Liver Failure on Expression and Function of Drug Transporters in the Kidney (Details in the Text).

Transporter	Cell Models (ref)	Animal Studies (ref)
P-gp		↑ NASH mice, rats [61]
BCRP		↔ NASH mice, rats [61]
MRP2	↑ RPTEC [62]	↑ CCl4—ALF rats [63] ↑ BDL rats [64] ↑ BDL rats [62] ↑ NASH mice, rats [61]
MRP3		↑ EHBR [65]
MRP4		↑ NASH mice, rats [61] ↓ BDL rats [66]
ASBT	↔ FPTC [67]	
OCT2		↓ NASH mice, rats [61]
OCT3		↔ NASH mice, rats [61]
OAT1		↔ NASH mice, rats [49] ↔ EHBR [68] ↑ BDL rats [69] ↓ BDL rats [70]
OAT3		↑ NASH mice, rats [61] ↑ EHBR [68] ↑ BDL rats [69]
OATP1		↑ ↔ NASH mice, rats [61]

RPTEC: human renal proximal tubular epithelial cells; NASH: nonalcoholic steatohepatitis; EHBR: Eisai hyperbilirubinemic rats; FPTC: fresh proximal tubular cells; ↓: decreased levels; ↑: increased levels; ↔: levels not changed.

## Abbreviations

ABC	ATP-binding cassette
AhR	aryl-hydrocarbon receptor
ALF	acute liver failure
ASBT	apical sodium-dependent bile acid transporter
BCRP	breast cancer resistance protein
BDL	bile duct ligation
CCl4	carbon tetrachloride
EHBR	Eisai hyperbilirubinemic rats
FPTC	fresh proximal tubular cells
FXR	farnesoid X receptor
HNF	hepatocyte nuclear factor
MATE	multidrug and toxin extrusion protein
MCT	monocarboxylic acid transporter
MDR	multidrug resistance
MRP	multidrug resistance protein
NASH	nonalcoholic steatohepatitis
OAT	organic anion transporter
OATP	organic anion transporting polypeptide
OCT	organic cation transporter
OCTN	organic cation/carnitine transporter
OST	organic solute transporter
PEPT	peptide transporter
PXR	pregnane X receptor
RPTEC	human renal proximal tubular epithelial cells;
SHP	small heterodimer partner
SLC	solute carrier
